# Remaining Useful Life Prognosis for Turbofan Engine Using Explainable Deep Neural Networks with Dimensionality Reduction

**DOI:** 10.3390/s20226626

**Published:** 2020-11-19

**Authors:** Chang Woo Hong, Changmin Lee, Kwangsuk Lee, Min-Seung Ko, Dae Eun Kim, Kyeon Hur

**Affiliations:** School of Electrical & Electronic Engineering, Yonsei University, 50 Yonsei-Ro Seodamun-Gu, Seoul 03722, Korea; spearw@yonsei.ac.kr (C.W.H.); lcmin@yonsei.ac.kr (C.L.); kwangsuklee@yonsei.ac.kr (K.L.); kms4634500@yonsei.ac.kr (M.-S.K.); daeeun@yonsei.ac.kr (D.E.K.)

**Keywords:** deep neural network, dimensionality reduction, explainable artificial intelligence, feature selection, prognostics and health monitoring, turbofan engine

## Abstract

This study prognoses the remaining useful life of a turbofan engine using a deep learning model, which is essential for the health management of an engine. The proposed deep learning model affords a significantly improved accuracy by organizing networks with a one-dimensional convolutional neural network, long short-term memory, and bidirectional long short-term memory. In particular, this paper investigates two practical and crucial issues in applying the deep learning model for system prognosis. The first is the requirement of numerous sensors for different components, i.e., the curse of dimensionality. Second, the deep neural network cannot identify the problematic component of the turbofan engine due to its “black box” property. This study thus employs dimensionality reduction and Shapley additive explanation (SHAP) techniques. Dimensionality reduction in the model reduces the complexity and prevents overfitting, while maintaining high accuracy. SHAP analyzes and visualizes the black box to identify the sensors. The experimental results demonstrate the high accuracy and efficiency of the proposed model with dimensionality reduction and show that SHAP enhances the explainability in a conventional deep learning model for system prognosis.

## 1. Introduction

The safety of turbofan engines in aircrafts should be pursued as the top priority. Owing to advances in materials and control technology, aircraft breakdowns due to defective components have been decreased and the management of turbofan engine malfunctions has become crucial for passenger safety. Successful maintenance of the turbofan engine helps ensure the aircraft’s reliability; hence, many advances have been made in terms of the maintenance and management of turbofan engines. In the early days, corrective maintenance, the concept of repairing in failure, was a commonly used maintenance strategy [1]. Since then, owing to the accumulation of operational experience, the approach has mainly developed into precautionary maintenance for equipment prone to failure. It has recently been expanded into condition-based maintenance (CBM), which involves performing maintenance at the optimal time, thereby apprehending the equipment’s condition. Through CBM, decision-makers can manage equipment economically and reliably in a way that minimizes the sum of repair and failure prevention costs. To properly perform CBM, it must be preceded by Prognostics and Health Management (PHM), which helps understand the current equipment status. PHM is a series of procedures that predict how long a piece of equipment can be used by obtaining and diagnosing equipment status information. Through PHM, optimal operation and maintenance plans can be established. The most important aim of performing PHM is to accurately predict the remaining useful life (RUL), which is the period from the current point before equipment failure [2]. Additionally, fault diagnosis and prognosis performance can be evaluated through Prognostic Horizon (PH), α-λ Performance, Relative Accuracy (RA), and Convergence Rate [3].

Three methods can be used for RUL prognoses: a physics-based approach, data-driven approach, and hybrid approach [4]. A physics-based approach can be used when a degradation model, such as fatigue crack growth, is available and significantly accurate [5,6]. However, considerable amounts of prior knowledge or measurement data are required, and the effect of noise must be considered. If there is no degradation model, the degradation characteristics can be predicted using a data-driven approach, which uses measurement data. If there is sufficient historical data, polynomials’ coefficients can be inferred to determine the correlation and causal relationship, and essential system information such as the RUL can be obtained. However, because it is not an accurate physics model, it has a disadvantage in that its accuracy is low. Lastly, the hybrid approach combines a physical-based approach and a data-based approach [7,8]. The prediction accuracy of the hybrid approach is improved by combining the polynomial advantages obtained from the data-driven method with the low error afforded by the physics-based method’s degradation model.

The data-driven method is mainly used to prognose the RUL of a turbofan engine. A turbofan engine comprises a compressor, a combustor, a turbine, and components that require numerous components. Various sensor data, such as the temperature, pressure, and speed of complex components, are collected to understand the condition of the turbofan engine. Owing to the complexity of the structure and the variety of sensors, a single degradation model cannot be used as a physics-based method to determine the turbofan engine’s condition. Recently, artificial neural networks (ANNs), especially deep neural networks (DNNs), have been used to predict RUL with high accuracy for nonlinear and complex systems. A DNN is an ANN that includes many hidden layers between the input layer and the output layer. In recent years, research has focused on obtaining better prediction results through various algorithms. To predict the turbofan engine’s RUL, among the machine learning algorithms, multilayer perceptron, support vector regression, and relevance vector regression are compared with the recent convolutional neural network (CNN) algorithm. In this case, the CNN algorithm showed higher accuracy [9], and higher accuracy was obtained by constructing a deep CNN model with five CNN layers [10]. Meanwhile, in a recurrent neural network (RNN), the long short-term memory (LSTM) algorithm was used to solve the gradient descent problem in which the learning effect decreased as the layer deepened during training [11]. The learning effect was improved using the bidirectional LSTM (Bi-LSTM) algorithm, which improved the performance by exchanging information in both directions of the input and output [12,13]. In addition, the RUL of the turbofan engine was predicted through a standard deep belief network (DBN) [14] and a semi-supervised deep architecture [15]. It was also shown that the accuracy improved when RUL was predicted by combining the CNN and LSTM algorithms [16,17,18].

The RUL prognosis of a turbofan engine has two aspects that need to be considered, apart from improving accuracy. First, several sensors are required to accurately predict the condition. As described above, a turbofan engine acquires several data from various sensors, such as the temperature, pressure, and speed data from the compressor, combustor, and turbine. This is because the data from all components are required to accurately diagnose the overall state of the turbofan engine. In this study, National Aeronautics and Space Administration (NASA) Commercial Modular Aero-Propulsion System Simulation (C-MAPSS) dataset’s 21 sensors were used [19]. However, in real turbofan engines, data are collected from hundreds of sensors. Excessive sensor data makes predictions difficult due to the amount and complexity of the data. Moreover, there is also a problem in terms of missing values or noise. The higher the data level, the greater the amount of information to be obtained; however, preprocessing is also needed to solve the “curse of dimensionality”. To mitigate the data level problem, the dimensionality reduction technique can be used. Dimensionality reduction involves transforming data from a high-dimension to low-dimension while maintaining critical characteristics of the original data. There are two methods of dimensionality reduction: feature selection and feature projection. Feature selection simplifies the model by removing redundant data or less-relevant variables. The simplification of the model simplifies the analysis, reduces training time, and prevents overfitting. Feature selection includes correlation analysis or regularization [20,21]. On the other hand, feature projection uses all the data to create a new combination of features. Combining original features into new features can include the existing characteristics while changing to a lower dimension than the original dimension. Methods such as principal component analysis or autoencoders are examples of this approach [22,23]. Good learning effects can be expected when using dimensionality reduction through feature extraction, while reducing computational costs and the amount of learning data.

In the prognosis of the RUL of a turbofan engine, there exists a lack of analysis of the prediction results. Most RUL predictions have the same problem. When considering the importance of monitoring the turbofan engine condition, it is fatal not to know which part has caused the engine to run out of life in maintaining the equipment. Many data-driven approaches have a limitation in that they lack the analysis of prediction results. In particular, when using a DNN, due to the “black box” problem, it is impossible to know how information is processed in the hidden layer. Decision-makers and domain experts cannot use predictive techniques correctly without an adequate explanation of the results. Recently, research on explainable artificial intelligence (AI) has been conducted to confirm the processes occurring inside the black box. Three approaches can be applied for explainable AI. The first is to extract features that can be explained through the trained model (model-specific explainability). The second is that the model itself can be explained as a decision tree (transparent model). The third method analyzes the cause by manipulating the input and viewing the output, without depending on the model (model agnostic explainability) [24]. Using these three approaches, it is possible to understand and determine the features that significantly affect the results when using a DNN. Accuracy and explainability have a trade-off in explainable AI. The ANN, especially DNN, has high accuracy in terms of predictions; however, its explanation is deficient because of the hidden layer. Therefore, to offer a prognosis of the RUL of a turbofan engine, it is essential to analyze the black box inside while using a DNN with high accuracy.

The proposed deep neural network accurately prognoses the RUL of a turbofan engine by organizing the 1D CNN, LSTM, and Bi-LSTM algorithms. The network combines the algorithms with different characteristics well and uses techniques to prevent overfitting, thereby affording higher accuracy than recently proposed approaches. Training and verification are performed using the C-MAPSS dataset by NASA. The NASA C-MAPSS sensor data have different correlations and are affected by noise. The dimensionality reduction technique is applied to reduce the complexity caused by several variables and improve accuracy. The accuracy achieved was compared by changing the input features using correlation analysis and regularization, which are feature selection techniques. In addition, Shapley additive explanation (SHAP) is adopted to identify variables that significantly influence the prediction results. SHAP’s visualization allows decision-makers to intuitively identify critical features that affect a turbofan engine’s RUL.

The remainder of this paper is organized as follows. Section 2 describes the 1D CNN, LSTM, and Bi-LSTM algorithms and network architecture techniques for improving accuracy by constructing a DNN. In addition, correlation analysis and regularization among the dimensionality reduction techniques, considering the turbofan engine’s characteristics, and the SHAP algorithm among explainable AI techniques for analyzing the prognosis results are described. Section 3 describes the experimental study and results, and the paper is concluded in Section 4.

## 2. Proposed Methodology

### 2.1. Deep-Stacked Convolutional Bi-LSTM Model

In this study, a deep-stacked convolutional Bi-LSTM model was constructed to provide a prognosis of a turbofan engine’s RUL. All three algorithms of CNN, LSTM, and Bi-LSTM were used. Each algorithm has different characteristics and advantages, and a deep-stacked model is constructed by appropriately combining the three algorithms. In addition, a residual network and dropout technique were used to prevent overfitting and increase accuracy.

#### 2.1.1. One-Dimensional CNN (1D CNN)

**Figure 1:**
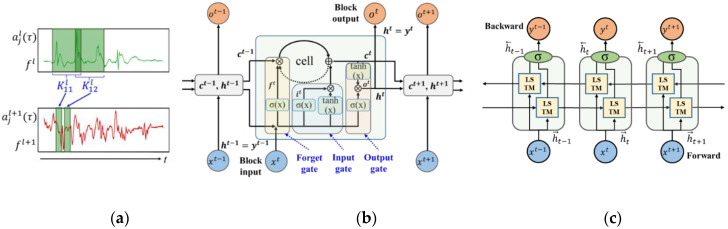
(**a**) Representation of one-dimensional convolutional neural network; (**b**) Long Short-Term Memory; (**c**) Bidirectional LSTM.

A CNN was initially developed to exploit 2D or 3D spatial feature information [25]. Inspired by the biological visual recognition process, CNNs are widely used in speech recognition, natural language processing, and image or video recognition [26]. The two main characteristics of a CNN are spatial shared weight and spatial pooling through convolution. The weight sharing structure reduces the complexity of the model and the number of weights. Raw input data are convoluted through several filters to generate features required for creating a feature map. The filter slides vertically and horizontally to extract data and determines the extent and precision of the information extracted by adjusting the filter size and stride size. Feature maps exploit essential local features through a pooling layer. The pooling layer can be applied to high-level problems such as image processing by extracting important local information from each feature map. However, some useful information is also filtered out; therefore, the accuracy may be lower than that of the RNN technique. The 1D CNN filter moves in a single direction, unlike the 2D CNN filter used for image processing. The 1D CNN has a relatively shallow architecture and can be used for time-series analyses of sensor data. In particular, the processing speed is faster than that of the 2D CNN, and the computational efficiency can be improved. The 1D CNN layer is expressed as

(1) $$a_{j}^{l+1}\left(\tau \right)=\;\sigma \left(\overset{f^{l}}{\underset{f=1}{\sum }}K_{jf}^{l}\left(\tau \right)\times \;a_{j}^{l}\left(\tau \right)+b_{j}^{l}\right)$$

where $a_{j}^{l}\left(\tau \right)$ represents the feature map *j* in layer *l*, $b_{j}^{l}$ is a bias vector of the feature map *j*, $f^{l}$ is the number of feature maps in layer *l*, and $K_{jf}^{l}$ is the kernel slide over the feature map *f* to generate the feature map j in the layer (*l* + 1). Layer *l* defines the input sensor data and σ is a nonlinear sigmoid function. The time *t* moves horizontally. The 1D CNN process is shown in Figure 1a.

#### 2.1.2. LSTM

LSTM is a more complex form of RNN. Existing RNNs have been used to process time-series data [27]; however, the RNN has a gradient descent problem in the training process. When the number of time steps is large, the initial weights are no longer constant, resulting in a decrease in learning ability. An advanced modified version of the RNN, called LSTM, was proposed to solve this problem [28]. The LSTM consists of an input gate, an output gate, a forget gate, and a memory block. It features long-term dependency because it incorporates a “memory” control block and a standard repeating layer. While the conventional RNN completely redefines cell states, LSTM can carefully remove or add information to cell states through gates. On determining that the information added to the cell is important, the system’s output performs one or more time steps on the memory contents. If the information is not essential, the system may determine to reset the memory unit’s contents by opening the forgetting gate. These three gates offer higher performance than general RNNs because even a multi-layered network can access previous information during training. As it has a vital feature of long-term dependency, LSTM has high accuracy in solving time-series sensor data prediction and natural language processing problems such as speech recognition and language translation. The structure of the LSTM network is shown in Figure 1b, and the equations are as follows:

(2) $$i^{t}=\sigma \left(W_{i}x^{t}+R_{i}y^{t-1}+p_{i}⊙c^{t-1}+b_{i}\right)$$

(3) $$f^{t}=\sigma \left(W_{f}x^{t}+R_{f}y^{t-1}+p_{f}⊙c^{t-1}+b_{f}\right)$$

(4) $$o^{t}=\sigma \left(W_{o}x^{t}+R_{o}y^{t-1}+p_{o}⊙c^{t-1}+b_{o}\right)$$

(5) $$c^{t}=z^{t}⊙i^{t}+c^{t-1}⊙f^{t}$$

(6) $$y^{t}=\;h(c^{t})⊙o^{t}$$

where $x^{t}$, $i^{t}$, $f^{t}$, $o^{t}$, $c^{t},$ and $z^{t}$ are input vectors at time *t*, input gate, forget gate, output gate, memory cell and block input, respectively. *W*, *R*, *p*, and *b* are the input weight, recurrent weight, peephole weight, and bias weight, respectively. The notation ⊙ denotes point-wise multiplication between two vectors, while σ and *h* are nonlinear activation functions. In LSTM, the cell state is divided into two parts: the long-term state *c(t)* and the short-term state *h(t)*. The cell state is regulated through three control gates along the state path: an input gate, a forgetting gate, and an output gate. The input gate *i(t)* controls the addition of information from the current output to the current long-term state *c(t)*, and the forgetting gate *f(t)* determine whether information from the previous long-term state *c(t−1)* should be removed. The output gate *o(t)* controls the formation of the current short-term state *h(t)* using the information of the current long-term state *c(t)*.

#### 2.1.3. Bi-LSTM

Traditional RNNs are suitable for processing sequential data but are trained only in the forward path. Bidirectional learning trains on both forward and reverse paths and uses the output for prediction as well. The LSTM can determine the storage and forgetting of information through the memory cell, and by deeply configuring this LSTM layer, it is possible to capture nonlinear characteristics. Bidirectional LSTM is an application of bidirectional learning to LSTM. If the vanilla LSTM only uses information going from the past to the future for learning, Bi-LSTM uses the information by independently calculating both the forward path and the reverse path. The output, which results from the flow of information, is also used for learning so that features are better extracted and have higher accuracy than the existing LSTM [29]. A Bi-LSTM network iterates layers from t = 1 to T in the forward direction and t = T to 1 in the reverse direction and updates the output. The formulation of the bidirectional LSTM layer can be defined as follows:

(7) $$y^{t}=\sigma \left({\vec{h}}_{t}⨁\;{\overset{\leftarrow }{h}}_{t}\right)$$

#### 2.1.4. Residual Network and Dropout Technique

In a DNN model, configuring the model through multiple layers is very important to improve performance. In theory, as the number of layers increases, the model extracts better features from the input data, but this can cause gradient descent problems. The residual network uses a method of connecting the previous layer’s output to the next layer’s output by skipping the layer block [30]. As the network gets deeper, the model learns feature information of a higher level; hence, information that has not been extracted before can be learned by combining the output of the previous layer and the output of the next layer. The residual network improves performance by merely adding connections using skipping layers without increasing network complexity. Using the residual network to construct a DNN for extracting time-series data features makes it easier to optimize and improve accuracy.

Dropout is a representative technique that can prevent overfitting at a low cost. The dropout technique temporarily deletes the connection between the input and output, including the unit in the network. This effect can be achieved by multiplying the output value of one unit by 0, and the same effect as learning multiple networks can be achieved by using the dropout technique. When several parameters are used in a DNN, overfitting may occur because of the units’ co-adaptation. Dropout networks require a longer time to learn on the same architecture but can significantly improve performance by preventing overfitting [31]. In particular, the dropout technique is not specific to a domain as a general technique. It improves the performance of neural networks in various applications such as speech recognition and image classification.

### 2.2. Dimensionality Reduction and Explainable Artificial Intelligence

In this section, we present some solutions to the problems that need to be solved for the practical application of the RUL prognosis. First, dimensionality reduction can mitigate the problem of excessive sensors. This study applies correlation analysis and regularization, which are feature selections among the dimensionality reduction techniques. Feature selection and feature projection methods exist for dimensionality reduction. However, feature selection, which is a method that does not damage the original sensor index, is more suitable for analyses of the predicted results, as discussed later. Second, to analyze the predicted result, among the explainable AI techniques, the SHAP technique is applied. The SHAP method analyzes the cause through the output without being dependent on the model and is suitable for DNNs with high accuracy. In addition, intuitive information can be provided to decision-makers through visualization of the analysis results.

#### 2.2.1. Correlation Analysis and Regularization

Feature selection differs from feature projection in that it maintains the index of the original sensor and performs during the preprocessing or pre-learning stage. By selecting the optimal subset, it is possible to reduce unnecessary computation and improve the accuracy of the model. There are three methods of feature selection: filter, wrapper, and embedded methods. The filter method is a method of selecting a feature before the learning algorithm. Pearson’s correlation coefficient analysis is a representative filter method for determining removal from a dataset based on the degree of correlation. The wrapper method detects the interaction between variables and evaluates the subset by finding the ideal combination of features through cross-validation. However, there is a risk of overfitting when the computational amount is large, and the number of features is small. The embedded method selects the optimal feature in the learning process of the model. Representatively, there is Ridge regression and least absolute shrinkage and selection operator (LASSO) techniques. In this study, the wrapper method that has the risk of overfitting is excluded to facilitate the deep learning model’s practical application. The effect on accuracy improvement is analyzed by applying the filter method and the embedded method. Through this technique, the complexity of sensor data can be reduced without complicated procedures. Pearson correlation analysis analyzes the bivariate correlation between two variables and is expressed as follows:

(8) $$r=\;\frac{\sum_{i=1}^{n}\left(X_{i}-\bar{X}\right)\left(Y_{i}-\bar{Y}\right)}{\sqrt{\sum_{i=1}^{n}{\left(X_{i}-\bar{X}\right)}^{2}}\sqrt{\sum_{i=1}^{n}{\left(Y_{i}-\bar{Y}\right)}^{2}}}$$

**Figure 2:**
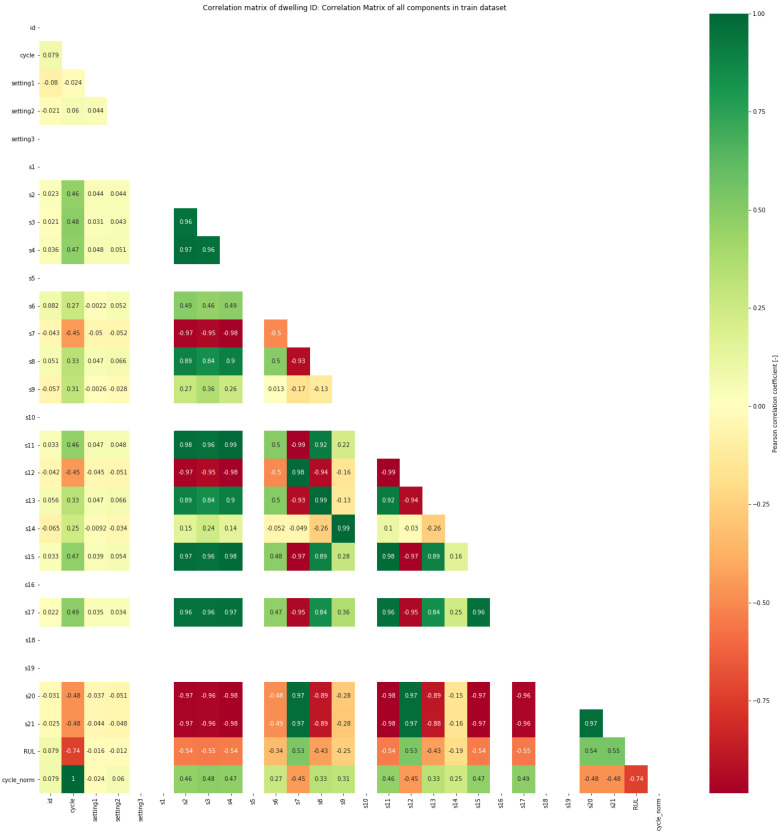
Correlation matrix heat map of the Commercial Modular Aero-Propulsion System Simulation (C-MAPSS) dataset.

The Pearson correlation coefficient *r* represents the correlation between two variables *X* and *Y* as a value between +1 and −1, and 0 indicates that there is no relationship. *n* is the number of samples, $X_{i}$ and $Y_{i}$ are the two sample points, and $\bar{X}$ and $\bar{Y}$ are the averages. Here, as the correlation coefficient between *X* and *Y* approaches 1, it implies that when one increases, the other tends to increase as well. The correlation matrix heat map expresses the correlation coefficient in terms of numbers and colors [32]. Figure 2 shows the correlation matrix heat map of the C-MAPSS dataset. Among the many input variables, one should focus on the correlation between sensor data from s1 to s21. Positive correlations are indicated by green, negative correlations are indicated by red, and no correlation is indicated by white. When the row and column numbers are the same, the correlation coefficient is always one because it is the correlation coefficient for itself; thus, the diagonal line’s upper right portion is omitted for convenience. In the correlation analysis, the high correlation between *X* and *Y* indicates their tendency to increase or decrease together. However, *Y* does not necessarily increase as *X* increases.

Regularization is one of the methods used to solve overfitting [33]. There are several sets of weights for predicting data in the network, and the more complex the model, the more likely it is to overfit. Therefore, to mitigate overfitting, the network’s complexity is limited so that the weight has a small value. It is called weight regulation, and there are three types of weight regulation. The first is LASSO (L1 regularization), which yields a regulation proportional to the weight’s absolute value. The second is Ridge (L2 regularization), which is proportional to the square of the weight. The last one is the Elastic Net, which is a combination of LASSO and Ridge. The formula for each regulation to add a penalty to the network’s loss function is as follows:

(9) $$\mathrm{LASSO}\;\mathrm{penalty}=\frac{1}{n}\overset{n}{\underset{i=1}{\sum }}\left\{L\left(y_{i},\;\hat{y_{i}}\right)+\frac{\lambda }{2}\left|w\right|\right\}$$

(10) $$\mathrm{Ridge}\;\mathrm{penalty}=\frac{1}{n}\overset{n}{\underset{i=1}{\sum }}\left\{L\left(y_{i},\;\hat{y_{i}}\right)+\frac{\lambda }{2}{\left|w\right|}^{2}\right\}$$

(11) $$\mathrm{Elastic}\;\mathrm{Net}\;\mathrm{penalty}=\frac{1}{n}\overset{n}{\underset{i=1}{\sum }}\left\{L\left(y_{i},\;\hat{y_{i}}\right)+\frac{\lambda }{2}\left|w\right|+\frac{\lambda }{2}{\left|w\right|}^{2}\right\}$$

where *n* is the size of the data, $L\left(y_{i},\;\hat{y_{i}}\right)$ is the existing loss function, λ is the regularization constant, and w is the weight. As λ approaches zero, the effect of regularization decreases. LASSO adds a penalty proportional to the weight’s absolute value, and Ridge adds a penalty proportional to the square of the weight. Elastic Net has the feature of adding all penalties imposed by LASSO and Ridge. LASSO has the characteristic of reducing the feature with little influence to 0, removing it, and selecting only the significant influencing features. Ridge makes the feature with little influence close to zero but does not converge it to zero. Therefore, the variance between features is reduced. Elastic Net performs the function of reducing both the number and variance of features.

#### 2.2.2. Explainable Artificial Intelligence (xAI)

Explainable AI emerged due to the lack of self-explanation, unlike the development of complex systems or artificial intelligence models. In the US Defense Advanced Research Projects Agency, a project called “Explainable Artificial Intelligence” aims at exploring the application of AI such that humans can understand the results of the solution [34]. Machine learning and artificial intelligence have an inherent “black box,” making it difficult to explain why AI makes specific decisions. To solve this problem, explainable AI should be separately applied, and there are three methods to do so. The first is to use a self-explanatory model. Models such as regression trees can be used for the self-explanatory approach [20]. The second is to extract features that can be explained through a trained deep learning model. The third one is to analyze the cause by evaluating the learning outcome regardless of the model, such as the local interpretable model-agnostic explanations (LIME) or the SHAP algorithm [35,36,37]. Unlike in the past, developments in computing power have made it easier to apply xAI, as the problem of interpretation has been solved. However, AI itself has a trade-off between accuracy and explanatory potential. Some models are easy to interpret for results, but such models are usually less accurate. Therefore, to give a prognosis for the turbofan engine’s RUL, it should be noted that it is essential to simultaneously explain the prediction results while achieving high prediction accuracy with a DNN. Decision-makers can develop long-term plans for maintenance or equipment replacement when simultaneously provided with reliable high prediction accuracy and interpretation of predictions. This study applies AI that can be explained in a model-independent method applicable to DNNs.

SHAP is a model agnostic explainable AI algorithm [38]. The Shapley value is a numerical indication of how much each participant contributed to creating the overall outcome. Each participant’s contribution can be expressed by measuring the increase or decrease in performance when that participant is excluded. SHAP uses Shapley values $\emptyset_{i}\left(v\right)$ to add based on the independence between features. It has the disadvantage that it requires a significant amount of time to derive the result because it calculates and visualizes each variable’s direction and size by simulating missing features. However, even when the model is complex and there are many variables, it is a significant advantage to show each variable’s influence over an intuitive figure. The SHAP formula is expressed as follows:

(12) $$\emptyset_{i}\left(v\right)=\;\underset{S\in N\left\{i\right\}}{\sum }\frac{\left|S\right|!\left(n-\left|S\right|-1\right)!}{n!}\left(v\left(S\cup \left\{i\right\}\right)-v\left(S\right)\right)$$

where $\emptyset_{i}$, *n*, *S*, v(S), and v(S∪{i}) are Shapley values for the *i*-th data, all participants, all sets except the *i*-th feature in the whole group, the contribution coefficient of the remaining subset except for the *i*-th feature, and the total contribution including the *i*-th feature, respectively. Shapley value decomposes the model’s output into each feature’s contribution and can also be expressed as a negative number. This case can be interpreted as indicating that the characteristics have a negative effect on the expected outcome through learning. SHAP results are displayed as intuitive figures, such as force plots or decision plots.

### 2.3. Proposed Deep Convolutional Bi-LSTM Network Model

**Figure 3:**
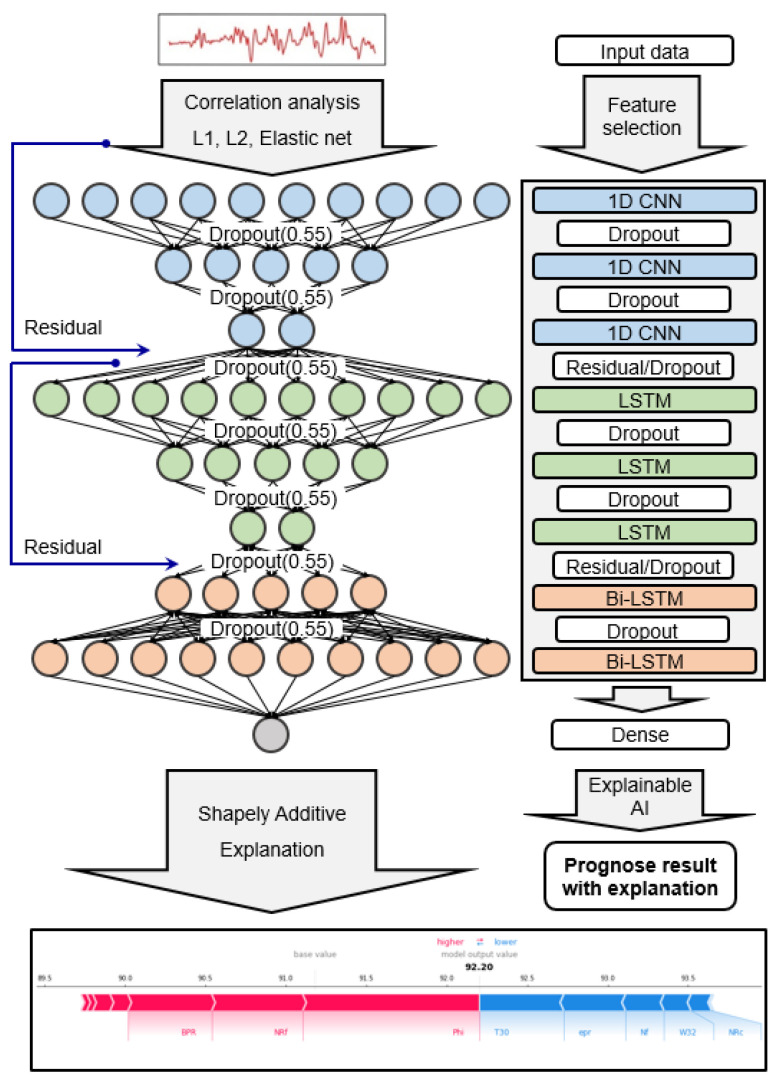
Proposed deep-stacked convolutional bidirectional LSTM model with dimensionality reduction and explainable artificial intelligence.

The proposed deep-stacked convolutional Bi-LSTM model with dimensionality reduction and explainable artificial intelligence is shown in Figure 3. In this study, a DNN was constructed using the CNN, LSTM, and Bi-LSTM algorithms. The three CNN layers extract spatial characteristics, the three LSTM layers extract temporal characteristics, and the two Bi-LSTM layers extract spatial and temporal characteristics through a bidirectional pass. In addition, the residual network and dropout technique were adapted to achieve accuracy and optimization. Moreover, to solve the practical problem regarding the prognosis of a turbofan engine’s RUL, correlation analysis, and regularization among dimensionality reduction techniques, and SHAP among explainable artificial intelligence techniques, were applied.

## 3. Experimental Study

The C-MAPSS dataset is typically used to give a prognosis for a turbofan engine’s RUL. The condition evaluation of the turbofan engine is a prerequisite for addressing equipment malfunction, maintenance, maintenance planning, and preventing potential loss of life. It is best to use the actual turbofan engine’s operating information, but it is complicated to obtain sensor information from equipment acquisition to failure. Therefore, NASA’s C-MAPSS dataset with several features was used for the experiment, which simulated the turbofan engine’s state. In the following section, we introduce the C-MAPSS dataset, hyperparameters, evaluation methods applied to the experiment, and the overall experiment’s flow chart.

### 3.1. C-MAPSS Dataset

**Figure 4:**
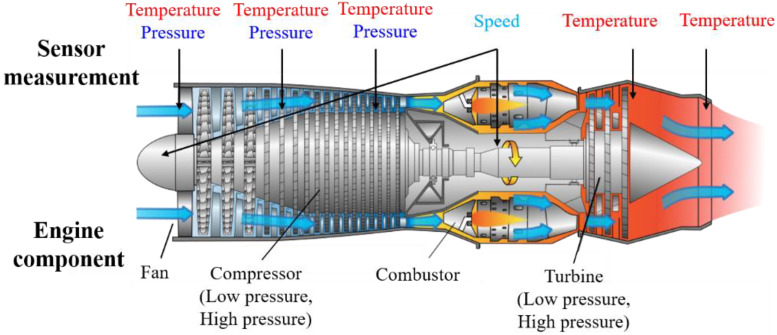
Diagram of the turbofan engine modules.

In this study, a turbofan engine’s RUL is given a prognosis using the C-MAPSS dataset FD001 of NASA. The turbofan engine consists of various components (fan, low pressure compressor, high pressure compressor, low pressure turbine, high pressure turbine, combustor, and nozzle), and the structure is shown in Figure 4. The information of each component, such as the temperature, pressure, speed, and air ratio, is acquired through sensors. The C-MAPSS dataset consists of four subsets, as shown in Table 1. Each subset is configured differently in terms of the number of engines, operating conditions, and failure types. The dataset also provides training data, test data, and RUL data, which are appropriate. The C-MAPSS dataset consists of 21 sensors that measure engine status and information from three operating settings; the list and corresponding physical meanings are presented in Table 2. The 24 multivariate time-series signals contain noise when depicting real data and illustrate the degradation until the engine fails as a time-series trajectory. Some engine sensor information indicates a direct deterioration trend for the engine, but some contain little or no information regarding performance degradation. In practical scenarios, domain experts and decision-makers can use information from sensors to monitor the turbofan engine’s condition and detect or rectify malfunctions. Furthermore, it is possible to predict performance degradation and RUL.

### 3.2. Hyperparameter Setup and Evaluation Metrics

The hyperparameters for training the deep-stacked convolutional Bi-LSTM model are shown in Table 3. The model consists of 8 layers, and the number of units is divided into four categories through correlation analysis. The input data were divided into mini batches of 200 samples, normalized to values between 0 and 1, and used for training. The dropout technique randomly excludes 55% of the units during training. For hyperparameter optimization, an adaptive moment estimation (Adam) algorithm, widely used with high performance, is used [39]. In the training phase, the model constitutes a final model by learning through a training set of 95% for 100 training data engines and then performs verification through a verification set consisting of 5%. The model then predicts the RUL for 100 engines with new test data. The total epochs were 200, and early stopping was applied to finish learning early when there was no learning improvement.

Root mean squared error (RMSE) is used to evaluate the trained final model’s RUL prognosis performance. There are many accuracy-based evaluation metrics used to evaluate regression models, including RUL estimation [40]. Both RMSE and mean squared error (MSE) are given the same penalty when the estimated RUL value is less than or greater than the actual RUL. The learning goal is to obtain 0, the smallest error for each engine’s RUL prognosis, which implies that the prediction result is the same as the actual RUL. MSE tends to be exaggerated because it deals with squared values. Therefore, the accuracy of the RMSE is compared with values reported in the literature. Additionally, mean absolute error (MAE) and mean absolute percentage error (MAPE) are described as a reference for the best case.

### 3.3. Flowchart of RUL Prognosis

**Figure 5:**
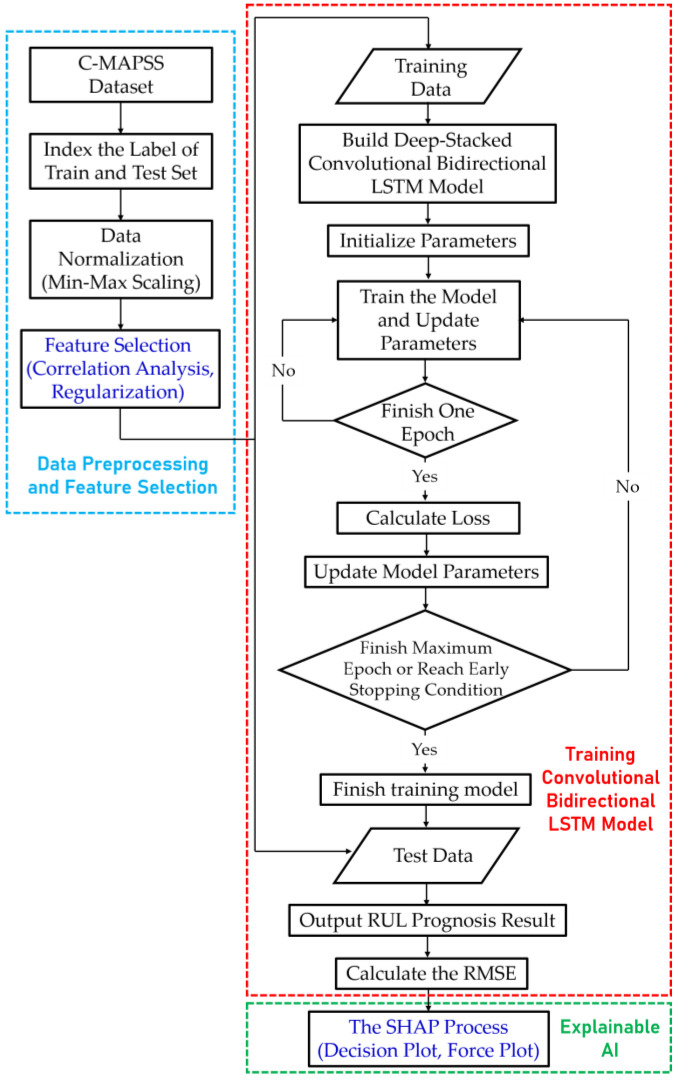
Flowchart of the experimental study.

The experiment’s overall process is shown in Figure 5, and the procedure for each step is as follows.

1. The C-MAPSS data are indexed and normalized into a training set and a test set. The data are then processed by correlation analysis and the feature selection technique of L1, L2, and Elastic Net.
2. The 21 time-series sensor data are divided into 200 mini batches and trained in the deep-stacked convolutional Bi-LSTM model. The model learns temporal and spatial characteristics with three 1D CNN layers, three LSTM layers, and two Bi-LSTM layers and uses the dropout technique and residual network to prevent overfitting and achieve high accuracy. Each time the epoch is completed on the first parameter, the loss is calculated and the parameter is updated. Learning ends when the maximum 200 epochs are completed or the early stopping condition is satisfied. After training is complete, the final model is determined, and the RUL and error are calculated by applying test data to this model.
3. The SHAP algorithm, an explainable AI technique, is applied to the predicted result. The explanation results can be visualized as a decision plot or force plot and used as analysis data.

## 4. Results and Discussion

### 4.1. Comparison with Literature

Table 4 shows a comparison with previous studies on the prognosis of the RUL of a turbofan engine using C-MAPSS data. The algorithm, network configuration, publication year, and predicted RMSE results of previous studies are summarized. Accuracy was improved by using advanced algorithms which were developed from machine learning techniques to CNNS to RNNS. In addition, accuracy was improved by deepening the network layer or using more than two algorithms. The proposed model combines CNN and RNN, and LSTM and Bi-LSTM are used together, among the RNN algorithms. The CNN layer easily exploits spatial features at the beginning of data entry. After the CNN layer, the LSTM layer learns temporal features and the previous layer’s spatial features. In the last Bi-LSTM layer, the turbofan engine’s RUL is given a prognosis by grasping the temporal and spatial characteristics through interactive learning. As the network layer becomes deeper, there is a possibility of improving the accuracy; however, overfitting also occurs. Therefore, the hyperparameters were fine-tuned, and a dropout technique and residual network were applied to avoid overfitting problems. Among the predictions of the RUL of turbofan engines for the last five years, the deep-stacked convolutional Bi-LSTM model proposed in this paper achieved higher accuracy. In particular, it should be noted that the RMSE of 10.41 is obtained, which is 25% and 14.9% lower RMSE than the study that predicted RUL by combining two or more algorithms [15,16].

### 4.2. Analysis of Feature Selection Effect

Feature selection during dimensionality reduction can improve performance by mitigating model complexity and overfitting problems by reducing the number or influence of input sensors. In a turbofan engine, as several sensors are used, the complexity problem can be solved by selectively removing and learning unnecessary or omissible sensors rather than using all sensors. In this study, the C-MAPSS FD001 dataset was divided into four categories through correlation analyses [41]: original (21 sensors in total), exclusion of sensors with a correlation of 0% (15 sensors in total), exclusion of sensors with correlation less than 30% (total of 14 sensors), and exclusion of sensors with correlation less than 60% (total of 12 sensors). For the same experimental conditions, the rest of the hyperparameters remain the same, except for the number of input sensors. In general, training on a set of features with low or little correlation yields inaccurate results. Table 5 shows that the best accuracy is obtained when sensor data with a correlation of less than 30% are excluded and learned. In excluding sensor data with a correlation of 0%, there was no significant difference compared to using the original dataset. However, in excluding sensor data with a correlation of less than 60%, the number of input sensors decreased, which adversely affected learning. It is evident that the accuracy is improved when some sensors are excluded through correlation analysis, considering a dataset’s characteristics.

**Figure 6:**
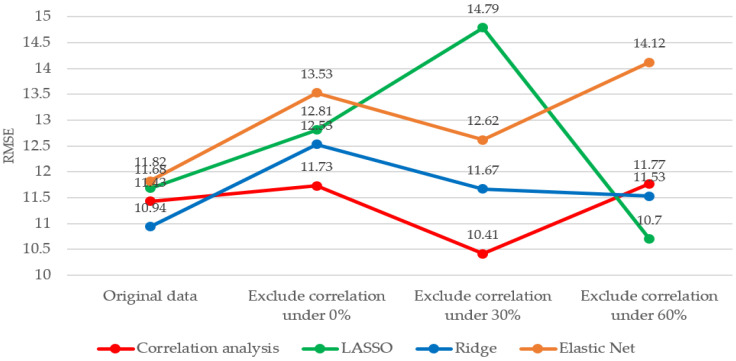
Comparison of RMSE for different feature selection methods.

The results of applying regularization were analyzed while changing the number of sensors according to the correlation analysis. A comparison of each regularization result is shown in Figure 6. When regularization was used on the original dataset, the accuracies of LASSO and Elastic Net were low; however, Ridge exhibited high accuracy. In excluding sensor data with a correlation of 0% and sensor data with less than 30%, LASSO, Ridge, and Elastic Net exhibited low accuracy. This result indicates that, although complexity can be reduced through regularization, the information necessary for learning is also removed. On excluding sensor data with a correlation of less than 60%, the accuracy was high when LASSO and Ridge were used. This shows that regularization played a role in mitigating the dataset’s multicollinearity problem. If the number of sensors is reduced through correlation analysis, the model’s complexity can be reduced, and the prediction accuracy can be improved. However, if excessive sensors are excluded, the features required for the prediction cannot be learned. This shows that the feature selection technique considering the number of features and missing values in the dataset could be optimized to obtain the best prediction result.

### 4.3. SHAP Analysis through Visualization

**Figure 7:**
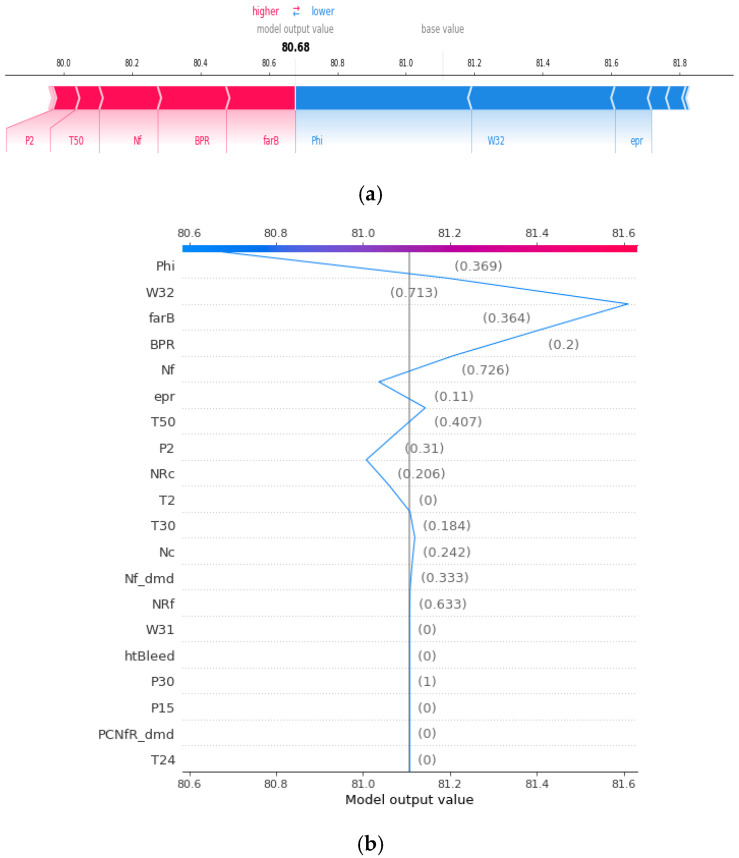
(**a**) SHAP force plot for engine No. 41 and (**b**) SHAP decision plot for engine No. 41.

**Figure 8:**
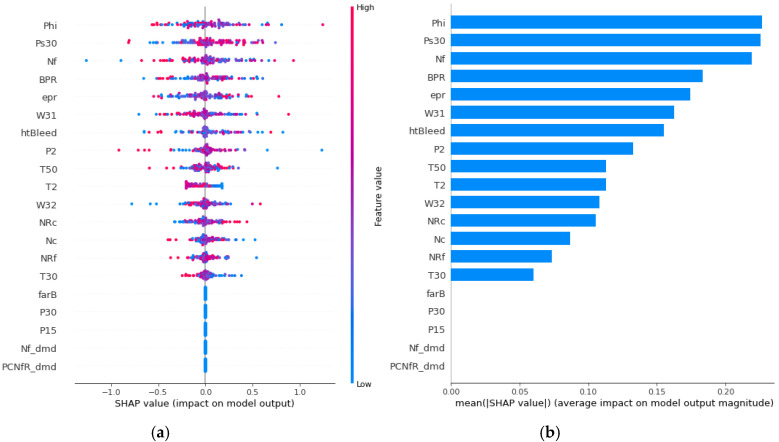
(**a**) SHAP summary plot (point) and (**b**) SHAP summary plot (absolute value).

**Figure 9:**
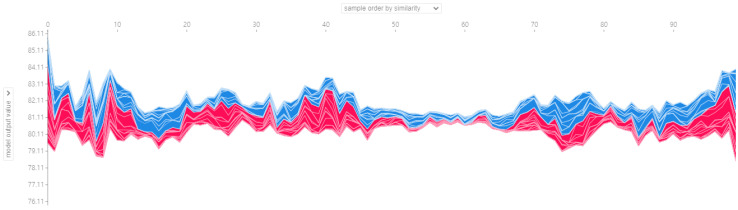
SHAP force plot of FD001 dataset.

After the training model gave the prognosis for the turbofan engine’s RUL, SHAP analysis was performed to explain the results. Because the training model uses a DNN, the SHAP is processed through DeepExplainer. DeepExplainer shows the contribution of each sensor to the predicted RUL as a score. A sensor with a positive Shapley value indicates that, by adequately managing the corresponding component, the turbofan engine can maintain its RUL suitably. However, a sensor with a negative Shapley value indicates that the deterioration of the corresponding component can reduce the entire turbofan engine’s life. Figure 7 shows each sensor’s contribution to the RUL prediction for engine 41 as a force plot and a decision plot. The red arrow represents a positive Shapley value, whereas the blue arrow denotes a negative Shapley value. The length of the arrow indicates the magnitude of the influence of each sensor. For the 41st turbofan engine, it can be seen that Phi (ratio of fuel flow to Ps30), W32 (LPT coolant bleed), and engine pressure ratio are sensors with a negative role in predicting RUL. In the decision plot, sensors representing negative Shapley values on the left and positive Shapley values on the right are listed according to the absolute value of RUL prediction’s degree of influence. Sensors that affect each engine can be intuitively identified through force plots and decision plots. The SHAP analysis for the entire test dataset is shown in the summary plot in Figure 8. Shapley values were applied to the entire dataset, and the impact was analyzed and displayed as a graph, listing the most influential sensors. In Figure 8a, the degree sensor’s influence on the RUL of each engine is expressed as points, and Figure 8b is expressed as the sum of the absolute values. Many outliers in Figure 8a indicate that each engine’s driving conditions and flight environments are significantly different and affected by noise. However, it should be noted that “phi” and “Ps30” have the most significant impact on RUL prediction, suggesting that HPC (high pressure compressor) management may affect the turbofan engine’s life. Lastly, the sensor’s Shapley value for each engine of the entire dataset can be seen in Figure 9.

The force plot in Figure 9 vertically accumulates and aligns each sensor’s Shapley values for 100 engines of FD001. Although listed according to similarity, it can be seen that the characteristics of the sensors for RUL prediction are different.

As a result of studying the influence of the turbofan engine dataset using the regression tree, the influence of sensor 7 (P30), sensor 12 (Phi), and sensor 21 (W32) was found to be high [20]. The SHAP analysis conducted in this study showed that sensor 12 (phi) has the most significant effect on RUL prediction, and sensor 21 (W32) has the 11th largest effect. However, it is necessary to consider that the RMSE is not as high as 36.08, obtained for the study using the regression tree. For appropriate analyses, it is necessary to use an accurate model with small errors before applying SHAP. It is also worth noting that the description of the SHAP is not absolute in actual engine management because the description of SHAP is based on prediction, even if it is analyzed using an accurate model. However, assuming that the description results have been analyzed by decision-makers or domain experts, they can be reflected in maintenance or equipment replacement plans, contributing to the longevity of turbofan engines.

## 5. Conclusions

In this study, the remaining useful life of a turbofan engine has been given a prognosis using a DNN that combines 1D CNN, LSTM, and Bi-LSTM. Feature selection methods and xAI techniques are adopted to solve the complexity and unobservability concerns of deep learning for its practical implementation. Case studies are conducted using the NASA turbofan engine dataset, and the results are compared with those of previous studies. The results of case studies and contributions are summarized as follows:

(1) The proposed deep learning model performs best in terms of accuracy in prognosis with reference to the benchmarked algorithms. Residual network and dropout techniques contribute toward the prevention of overfitting.
(2) During the feature selection, correlation analysis and regularization are employed to remove irrelevant input sensors or weights. The regularized model can significantly reduce complexity, while maintaining high accuracy.
(3) Use of xAI techniques helps identify primary sensors (i.e., components) influential to the prognosis. The xAI helps specify the “black box” deep learning model, which is surely beneficial for preventive and predictive engine maintenance.

## Figures and Tables

**Table 1:** NASA C-MAPSS dataset.

Dataset	FD001	FD002	FD003	FD004
Number of engines	100	260	100	249
Number of training samples	20,631	53,579	24,270	61,249
Number of test samples	100	259	100	248
Number of the data column	26	26	26	26
Average life span (cycles)	206	206	247	245
Operating conditions	1	6	1	6
Fault conditions	1	1	2	2

**Table 2:** List and physical meanings of the C-MAPSS dataset.

Sensor Number	Symbol	Description	Units	Trend
1	T2	Total temperature at fan inlet	°R	~
2	T24	Total temperature at LPC outlet	°R	↑
3	T30	Total temperature at HPC outlet	°R	↑
4	T50	Total temperature at LPT outlet	°R	↑
5	P2	Pressure at fan inlet	psia	~
6	P15	Total pressure in bypass-duct	psia	~
7	P30	Total pressure at HPC outlet	psia	↓
8	Nf	Physical fan speed	rpm	↑
9	Nc	Physical core speed	rpm	↑
10	epr	Engine pressure ratio	--	~
11	Ps30	Static pressure at HPC outlet	psia	↑
12	Phi	Ratio of fuel flow to Ps30	pps/psi	↓
13	NRf	Corrected fan speed	rpm	↑
14	NRc	Corrected core speed	rpm	↓
15	BPR	Bypass ratio	--	↑
16	farB	Burner fuel-air ratio	--	~
17	htBleed	Bleed enthalpy	--	↑
18	Nf_dmd	Demanded fan speed	rpm	~
19	PCNfR_dmd	Demanded corrected fan speed	rpm	~
20	W31	HPT coolant bleed	lbm/s	↓
21	W32	LPT coolant bleed	lbm/s	↓

**Table 3:** Hyperparameters for deep-stacked convolutional bidirectional LSTM model.

**Architecture Hyperparameters**					
**Number of Hidden Layers**	**Number of Units**	**Normalization**	**Activation**	**Mini Batch**	**Regularization**
8(3, 3, 2)	128–64–22(15, 14, 13)–128–64–22(15, 14, 13)–256–512	Min-Max Scaling	ReLu	200	Dropout (0.55)
**Optimization Hyperparameters**					
**Algorithm**	**Validation**	**Loss**	**Epochs**	**Decay**	**Learning Rate**
Adam	5% of train data	RMSE	Early stopping (Max. 200)	0	0.0001

**Table 4:** Comparison of the prognosis RMSE results with other algorithms.

Method	Description	Years	RMSE
MLP [8]	Multilayer Perceptron	2016	37.56
SVR [8]	Support Vector Regression	2016	20.96
RVR [8]	Relevance Vector Regression	2016	23.80
CNN [8]	2 CNN	2016	18.45
LSTM [10]	2 LSTM	2017	16.14
DBN [13]	Deep Belief Network	2017	15.21
BLSTM [11]	2 Bi-LSTM	2018	14.26
RNN [9]	5 Recurrent layers	2018	13.44
DCNN [9]	5 CNN	2018	12.61
Bi-LSTM [12]	2 Bi-LSTM	2018	13.65
Semi-Supervised [14]	RBM + 2 LSTM	2019	12.56
HDNN [15]	3 CNN/3 LSTM Hybrid	2019	13.02
DAG [16]	(CNN/LSTM Hybrid) + LSTM	2019	11.96
Proposed method	3 CNN + 3 LSTM + 2 Bi-LSTM	2020	10.41

**Table 5:** RUL prognosis RMSE result with feature selection method.

Algorithm	Original Data (21 Sensors)	Exclude Correlation under 0% (6 Sensors Removed)	Exclude Correlation under 30% (7 Sensors Removed)	Exclude Correlation under 60% (9 Sensors Removed)	Average RMSE
Correlation Analysis	11.43	11.73	10.41 (MSE 108.40, MAE 7.36, MAPE 14.05)	11.77	11.34
LASSO	11.68	12.81	14.79	10.70	12.50
Ridge	10.94	12.53	11.67	11.53	11.67
Elastic Net	11.82	13.53	12.62	14.12	13.02

## References

[1] Pintelon L., Parodi-Herz A. (2008). Maintenance: An evolutionary perspective. Complex System Maintenance Handbook.

[2] Lee J., Jin C., Liu Z., Ardakani H.D. (2017). Introduction to data-driven methodologies for prognostics and health management. Probabilistic Prognostics and Health Management of Energy Systems.

[3] Djeziri M.A., Benmoussa S., Zio E. (2020). Review on Health Indices Extraction and Trend Modeling for Remaining Useful Life Estimation. Artificial Intelligence Techniques for a Scalable Energy Transition.

[4] Kim N.-H., An D., Choi J.-H. (2016). Prognostics and Health Management of Engineering Systems: An Introduction.

[5] Byington C.S., Watson M., Edwards D., Stoelting P. A model-based approach to prognostics and health management for flight control actuators. Proceedings of the 2004 IEEE Aerospace Conference Proceedings (IEEE Cat. No. 04TH8720).

[6] Oh H., Azarian M.H., Pecht M., White C.H., Sohaney R.C., Rhem E. Physics-of-failure approach for fan PHM in electronics applications. Proceedings of the 2010 Prognostics and System Health Management Conference.

[7] Zhang H., Kang R., Pecht M. A hybrid prognostics and health management approach for condition-based maintenance. Proceedings of the 2009 IEEE International Conference on Industrial Engineering and Engineering Management.

[8] Sun T., Xia B., Liu Y., Lai Y., Zheng W., Wang H., Wang W., Wang M. (2019). A novel hybrid prognostic approach for remaining useful life estimation of lithium-ion batteries. Energies.

[9] Babu G.S., Zhao P., Li X.-L. (2016). Deep convolutional neural network based regression approach for estimation of remaining useful life. Lect. Notes Comput. Sci..

[10] Li X., Ding Q., Sun J.-Q. (2018). Remaining useful life estimation in prognostics using deep convolution neural networks. Reliab. Eng. Syst. Saf..

[11] Zheng S., Ristovski K., Farahat A., Gupta C. Long short-term memory network for remaining useful life estimation. Proceedings of the 2017 IEEE International Conference on Prognostics and Health Management (ICPHM).

[12] Wang J., Wen G., Yang S., Liu Y. Remaining useful life estimation in prognostics using deep bidirectional lstm neural network. Proceedings of the 2018 Prognostics and System Health Management Conference (PHM-Chongqing).

[13] Zhang A., Wang H., Li S., Cui Y., Liu Z., Yang G., Hu J. (2018). Transfer learning with deep recurrent neural networks for remaining useful life estimation. Appl. Sci..

[14] Zhang C., Lim P., Qin A.K., Tan K.C. (2016). Multiobjective deep belief networks ensemble for remaining useful life estimation in prognostics. IEEE Trans. Neural Netw. Learn. Syst..

[15] Ellefsen A.L., Bjørlykhaug E., Æsøy V., Ushakov S., Zhang H. (2019). Remaining useful life predictions for turbofan engine degradation using semi-supervised deep architecture. Reliab. Eng. Syst. Saf..

[16] Al-Dulaimi A., Zabihi S., Asif A., Mohammadi A. (2019). A multimodal and hybrid deep neural network model for remaining useful life estimation. Comput. Ind..

[17] Li J., Li X., He D. (2019). A directed acyclic graph network combined with CNN and LSTM for remaining useful life prediction. IEEE Access.

[18] Hong C.W., Lee K., Ko M.-S., Kim J.-K., Oh K., Hur K. Multivariate Time Series Forecasting for Remaining Useful Life of Turbofan Engine Using Deep-Stacked Neural Network and Correlation Analysis. Proceedings of the 2020 IEEE International Conference on Big Data and Smart Computing (BigComp).

[19] Saxena A., Goebel K., Simon D., Eklund N. Damage propagation modeling for aircraft engine run-to-failure simulation. Proceedings of the 2008 International Conference on Prognostics and Health Management.

[20] Yu L., Liu H. Feature selection for high-dimensional data: A fast correlation-based filter solution. Proceedings of the 20th International Conference on Machine Learning (ICML-03).

[21] Shi Y., Miao J., Wang Z., Zhang P., Niu L. (2018). Feature Selection with l_2,1−2_ Regularization. IEEE Trans. Neural Netw. Learn. Syst..

[22] Lasheras F.S., Nieto P.J.G., de Cos Juez F.J., Bayón R.M., Suárez V.M.G. (2015). A hybrid PCA-CART-MARS-based prognostic approach of the remaining useful life for aircraft engines. Sensors.

[23] Yu W., Kim I.Y., Mechefske C. (2019). Remaining useful life estimation using a bidirectional recurrent neural network based autoencoder scheme. Mech. Syst. Signal Process..

[24] Arrieta A.B., Díaz-Rodríguez N., Del Ser J., Bennetot A., Tabik S., Barbado A., García S., Gil-López S., Molina D., Benjamins R. (2020). Explainable Artificial Intelligence (XAI): Concepts, taxonomies, opportunities and challenges toward responsible AI. Inf. Fusion.

[25] LeCun Y., Bottou L., Bengio Y., Haffner P. (1998). Gradient-based learning applied to document recognition. Proc. IEEE.

[26] Gu J., Wang Z., Kuen J., Ma L., Shahroudy A., Shuai B., Liu T., Wang X., Wang G., Cai J. (2018). Recent advances in convolutional neural networks. Pattern Recognit..

[27] Bengio Y., Simard P., Frasconi P. (1994). Learning long-term dependencies with gradient descent is difficult. IEEE Trans. Neural Netw..

[28] Hochreiter S., Schmidhuber J. (1997). Long short-term memory. Neural Comput..

[29] Schuster M., Paliwal K.K. (1997). Bidirectional recurrent neural networks. IEEE Trans. Signal Process..

[30] He K., Zhang X., Ren S., Sun J. Deep residual learning for image recognition. Proceedings of the IEEE Conference on Computer Vision and Pattern Recognition.

[31] Srivastava N., Hinton G., Krizhevsky A., Sutskever I., Salakhutdinov R. (2014). Dropout: A simple way to prevent neural networks from overfitting. J. Mach. Learn. Res..

[32] Friendly M. (2002). Corrgrams: Exploratory displays for correlation matrices. Am. Stat..

[33] Ying X. (2019). An overview of Overfitting and its solutions. J. Phys. Conf. Ser..

[34] Gunning D. Explainable Artificial Intelligence (xai). https://www.cc.gatech.edu/~alanwags/DLAI2016/(Gunning)%20IJCAI-16%20DLAI%20WS.pdf.

[35] Ponn T., Kröger T., Diermeyer F. (2020). Identification and Explanation of Challenging Conditions for Camera-Based Object Detection of Automated Vehicles. Sensors.

[36] Dindorf C., Teufl W., Taetz B., Bleser G., Fröhlich M. (2020). Interpretability of input representations for gait classification in patients after total hip arthroplasty. Sensors.

[37] García M.V., Aznarte J.L. (2020). Shapley additive explanations for NO2 forecasting. Ecol. Inform..

[38] Lundberg S.M., Lee S.-I. (2017). A unified approach to interpreting model predictions. NIPS’17: Proceedings of the 31st International Conference on Neural Information Processing Systems, Long Beach, CA, USA, 4–9 December 2017.

[39] Kingma D.P., Ba J. Adam: A Method for Stochastic Optimization. https://arxiv.org/pdf/1412.6980.pdf.

[40] Saxena A., Celaya J., Balaban E., Goebel K., Saha B., Saha S., Schwabacher M. Metrics for evaluating performance of prognostic techniques. Proceedings of the 2008 International Conference on Prognostics and Health Management.

[41] Schober P., Boer C., Schwarte L.A. (2018). Correlation coefficients: Appropriate use and interpretation. Anesth. Analg..

